# Randomized controlled trials in frontotemporal dementia: cognitive and behavioral outcomes

**DOI:** 10.1186/2047-9158-3-12

**Published:** 2014-06-05

**Authors:** Justin B Miller, Sarah J Banks, Gabriel C Léger, Jeffrey L Cummings

**Affiliations:** 1 Cleveland Clinic, Lou Ruvo Center for Brain Health, 888 W. Bonneville Ave, Las Vegas, Nevada 89106, USA

**Keywords:** Frontotemporal dementia, Primary endpoints, Secondary endpoints, Randomized controlled trial, Methods

## Abstract

Progress has been made in understanding the genetics and molecular biology of frontotemporal dementia (FTD). Targets for intervention have been identified, therapies are being developed, and clinical trials are advancing. A major challenge for FTD research is that multiple underlying pathologies can be associated with heterogeneous phenotypes. The neuropsychological profiles associated with FTD spectrum disorders often include executive dysfunction, language impairments and behavioral disturbance. Behavioral variant FTD is characterized by an initial presentation of changes in personality, behavior and/or emotion, which are often difficult to objectively capture using traditional neuropsychological measures. The two principal language variants of FTD are Progressive Nonfluent Aphasia (PNFA) with predominant agrammatic/non-fluent impairments and Semantic Dementia (SD) with semantic impairments and visual agnosia. Selection of appropriate endpoints for clinical trials is critical to ensure that the measures are adequately sensitive to detect change, yet specific enough to isolate signal from noise, and acceptable to regulatory agencies. Given the anticipated potential for small effect sizes, measures must be able to identify small incremental changes over time. It is also imperative that the measures provide adequate coverage of the constructs or behaviors of interest. Selected outcome measures should be suitable for repeat administration, yet relatively robust to practice effects to ensure that observed changes reflect true signal variance and not residual effects due to repeated measurement or poor reliability. To facilitate widespread adoption as an endpoint, measures should be readily accessible. We provide several examples of potential global, composite, and individual cognitive measures, as well as behavioral measures promising for FTD trials. Development and application of appropriate trial outcomes is critically important to success in advancing new treatments for FTD patients.

## 

Frontotemporal dementia (FTD) is a clinically and biologically diverse neurodegenerative disease that rivals the prevalence of Alzheimer’s disease (AD) in adults younger than 65 [1]. A major challenge for FTD research is that there are multiple underlying pathologies [2,3], and any of the identified pathologies can be associated with heterogeneous phenotypes depending upon the lesion type, load, and distribution [4,5]. Classifications of FTD are evolving based on genotype, protein abnormality, and phenotype. The neuropsychological profile associated with FTD spectrum disorders often includes executive dysfunction and language impairments. Behavioral variant frontotemporal dementia (bvFTD) is characterized by an initial presentation of changes in personality, behavior and/or emotion which are often difficult to objectively capture using traditional neuropsychological measures. There are two principal language variants associated with FTD: Primary Progressive Nonfluent Aphasia (PPNA) with predominant agrammatic/non-fluent impairments or Semantic Dementia (SD) with fluent verbal output and semantic impairments [6]. A third language variant, Logopenic progressive aphasia, is occasionally associated with FTD, although most cases with aphasia of the logopenic type are due to AD [6].

Improved understanding of the neurobiology of FTD has led to the identification of candidate therapies that address the underlying pathophysiology associated with this group of disorders [7]. Clinical trials are anticipated as promising agents are introduced to human populations to assess efficacy. Given the phenotypic diversity of FTD, selection of appropriate endpoints for clinical trials is challenging and making good choices is critical to ensure that the trial measures are adequately sensitive to detect change, yet specific enough to isolate signal from noise, and acceptable to regulatory agencies (i.e., Food and Drug Administration, FDA; European Medicines Agency, EMA). The primary aim of this paper is to discuss considerations for identification and selection of appropriate cognitive and behavioral endpoints (e.g., domains of function) for use in clinical trials. It is not our intent to be prescriptive about specific measures or endpoints to employ, but to generate recommendations and identify critical factors to consider during trial planning to facilitate selection of neuropsychological endpoints. There are a number of biomarkers that should also be considered for use in randomized clinical trials (RCTs) for FTD, however, discussion of such measures is beyond the scope and aims of this paper. Here, we restrict our emphasis to the cognitive and behavioral phenotypes, relevant to selecting outcomes for RCTs.

## FDA Recommended Outcomes in RCTs

In order to promote uniformity across drug development for dementia disorders, the United States FDA mandates several essential outcome types that must be included in dementia-related trials. Although the FDA does not have requirements for specific tests or measures that need to be included, nonbinding recommendations are made regarding the domains to be assessed. In AD trials --- which function as a guide to how to conduct FTD trials --- the FDA requires dual outcomes: a measure of the core cognitive features of the disorder and a global or functional measure to determine the clinical meaningfulness of any therapeutic benefit [8]. Often based on clinician ratings, global measures attempt to provide an overall quantitative estimate of cognition, behavior, and daily functioning and are frequently used as a co-primary endpoint [9]. Examples of commonly used global measures in AD trials include the Clinical Dementia Rating (CDR) [10] and the Clinicians’ Interview-Based Impression of Change (CIBIC) [9]. An alternative indicator of clinical meaningfulness is the use of a measure of activities of daily living such as the Alzheimer’s Disease Cooperative Study (ADCS) Activities of Daily Living (ADL) scale [11] or the Disability Assessment for Dementia (DAD) [12].

These global or functional measures are complemented by a measure of the core cognitive components of the dementia syndrome. In AD, the Alzheimer’s Disease Assessment Scale - Cognitive Portion (ADAS-Cog) [13] is the most commonly used neuropsychological assessment. This tool, however, lacks executive measures, emphasizes capture of the memory impairment characteristic of AD and does not explore language in depth, limiting its usefulness for FTD clinical trials. Alternative measures sensitive to the specific abnormalities found in FTD are needed.

Secondary outcome measures are commonly used in dementia trials to assess behavioral [14] and economic outcomes [15]. These secondary outcomes provide additional insight into drug effects but are not included in the package insert description of an approved agent.

Although FTD has known and identifiable pathologies and several potential biomarkers [16], use of biomarkers as a surrogate for clinical benefit is currently not available in dementia syndromes [17]. Until such evidence exists, measures of cognition will remain the central marker of change and clinical benefit.

## Current summary of randomized clinical trials in FTD

There have been relatively few randomized clinical trials (RCTs) in FTD, and those that have been conducted have been small and often inconclusive, particularly with regard to cognition. A review of RCTs published in the last decade indicates that several existing pharmacological interventions may be beneficial for reducing behavioral disturbances in FTD, however, none of the reviewed studies yielded any benefit for improving cognition [18] and some have shown undesirable effects [7,19]. A summary of the endpoints reported in the published trials is presented in Table 1.

**Table 1:** Summary of published endpoints in randomized controlled trials in frontotemporal dementia

**Study**	**Sample size**	**Global endpoints**	**Cognitive endpoints**	**Behavioral endpoints**
Moretti, R. et al. [20]	16	---	MMSE, Ten Point Clock Test, Proverb Interpretation Tasks, Stroop Test	NPI, CIRS, CSDD, BEHAVE-AD
Deakin, J.B. et al. [21]	10	---	CANTAB*, Verbal fluency, Digit Span	NPI, CBI
Lebert, F. [22]	26	CGI-I	MMSE	NPI
Rahman, S. [23]	8	---	NART, MMSE, CANTAB**, Cambridge Gamble Task	---
Huey, E.D. [24]	8	---	RBANS	NPI
Kertesz, A. [25]	36	CGI-S, CGI-I	WAB, MMSE, DRS	FBI, NPI, ADLS
Vercelletto, M. [19]	49	CIBIC+	MMSE, DRS	NPI, FBI, DAD, ZBI
Boxer, A.L. [7]	81	CGI-C	CVLT, fluency, BNT, Trail Making test, Digit Backwards, Digit symbol	NPI
Jesso, S. [26]	20	---	Emotion recognition, emotion processing, Theory of Mind task	NPI, FBI

*ADLS*, alzheimer’s disease cooperative study—activities of daily living scale; *BEHAV-AD*, behavioral pathology in alzheimer’s disease rating scale; *BNT*, Boston naming test; *CBI*, Cambridge behavioral inventory; *CGI-C*, clinical global impression of change; *CGI-I*, clinical global impression of improvement; *CGI-S*, clinical global impression of severity; *CIBIC+*, clinician’s interview-based impression of change plus caregiver input; *CIRS*, clinical insight rating scale; *CSDD*, cornell scale for depression in dementia; *CVLT*, California verbal learning test; *DAD*, disability assessment for dementia; *DRS*, dementia rating scale, *FBI*, frontal behavioral inventory; *MMSE*, mini mental status exam; *NART*, National test of adult reading; *NPI*, neuropsychiatric inventory; *RBANS*, repeatable battery for the assessment of neuropsychological status; *WAB*, Western Aphasia battery; *ZBI*, Zarit burden inventory.

*(immediate and delayed pattern recognition, spatial recognition, spatial span, spatial working memory, visual discrimination learning/attentional set shifting, decision-making “gamble,” and paired associates learning).

**(pattern recognition memory, spatial recognition memory, spatial span, spatial working memory, and intradimensional (ID)/extradimensional (ED) attentional-set shifting, and Tower of London test of spatial planning).

Among the reviewed trials, the Clinical Global Impression (CGI) and its subscales specific to change (CGI-C), improvement (CGI-I) and severity (CGI-S) was used in 3 trials [7,22,25], and the CIBIC with caregiver input (CIBIC+) was used in one as a global measure [19]. Assessment of cognition was much more variable across the trials, with little evidence of uniformity in either domain coverage or assessment approach. Memory and executive functioning were the most commonly assessed domains. Three studies assessed episodic memory explicitly via subscales of composite batteries (e.g., Dementia Rating Scale; DRS [27]; Repeatable Battery for the Assessment of Neuropsychological Status; RBANS [28]) and six of nine studies evaluated some component of executive functioning, though there was no standard approach. The Mini-Mental Status Exam (MMSE) [29] was the most frequently administered cognitive measure, with use in five of nine trials. Several studies employed a battery of cognitive tests, including the Cambridge Neuropsychological Test Automated Battery (CANTAB) and the DRS, which were the second most frequently used measures, appearing in two trials each [19,21,23,25]. The RBANS was employed in one trial [24]. The diversity of approaches observed in these trials suggests that a consensus has not been reached on how best to assess FTD spectrum disorders in RCTs.

Much greater uniformity was apparent across trials with regard to behavioral endpoints, and most trials employed multiple behavioral endpoints. The Neuropsychiatric Inventory (NPI) [14] was the most frequently employed, appearing in eight of nine studies. The extent to which findings of behavioral improvement across trials is related to greater uniformity in assessment approaches remains unclear, though greater consistency would minimize superfluous variance related to methods.

## Limitations of the existing literature

Although there are myriad reasons why a trial could fail, one possible explanation for the lack of significant findings may relate to the endpoint selection. Within the field of neuropsychology, there is a relative lack of consensus regarding operationalization of cognitive constructs and selection of measures to quantify those constructs, with many different tests currently being used in research and clinical applications see [30,31] for review. The result is that the same construct has been defined and measured in multiple ways, using different tests that do not necessarily overlap. One immediate consequence of this variability is the introduction of unique method variance to outcomes research due to the use of tests with varying psychometric properties (e.g., standard error of measurement, reliability), which potentially masks treatment effects, inflates Type I and Type II measurement error, and hinders large-scale aggregation of data for meta-analytic study. The lack of evidence for cognitive improvement in a RCT may also be due to selection of insensitive measures. In the early phases of the disease, the changes in cognition may be so subtle that the measures employed lacked adequate sensitivity to small magnitudes of change.

One approach to enhancing uniformity and facilitating use of appropriate measures is to promote convergence among investigators toward common methods and data elements (e.g., NIH Toolbox, The Cognitive Atlas, Patient Reported Outcome Measurement Information System [PROMIS]), particularly for those tools used in clinical trials. Although the trial performance characteristics are unknown, the Uniform Data Set (UDS) for FTLD is one example of a brief cognitive battery that has been developed and successfully deployed to create uniformity among assessments at Alzheimer’s Disease Centers [32]. The NIH EXAMINER is a battery targeting brief assessment of executive functioning and social cognition, specifically for use in clinical trials. It has shown promise for the assessment of executive functions [33,34] and if acceptable performance characteristics in clinical trials can be demonstrated, its adoption would facilitate measurement standardization.

## What makes a good endpoint?

During the planning phase of a controlled trial, selection of appropriate measures is crucial, and there are multiple factors to consider in addition to FDA or EMA requirements. Given the potential for small effect sizes, measures must be able to identify small incremental changes over time by employing a metric that is fine enough to detect such changes. For example, using a measure with a binary metric (e.g., “normal” vs. “impaired”) may be too coarse and risk missing more subtle degrees of change. It is also imperative that the measures provide adequate coverage of the constructs or behaviors of interest, sampling over the entire range of possible outcomes in order to minimize limitations imposed by statistical distributions (i.e., ceiling and floor effects). Using measures that have a level of difficulty so low that baseline assessments result in a preponderance of scores falling at or near the ceiling is inappropriate, as such a distribution of scores allows for change in only one direction (i.e., decline). Measures also cannot be so difficult that the distribution of obtained scores is skewed towards the floor, for similar reasons. Additionally, by selecting measures with inadequate coverage, or too small a range of possible measurements, the risk of generating skewed data is increased.

Outcome measures should also be suitable for repeat administration, yet relatively robust to practice effects to ensure that observed changes reflect true signal variance and not residual effects due to repeated measurement (i.e., practice effects) or poor reliability. The inherent nature of a randomized controlled trial results in multiple assessments over the course of the trial and there are several methods to help account for practice effects. Some measures, however, are more vulnerable than others. For example, use of the Wisconsin Card Sorting Test [35], while useful in some clinical contexts, is particularly susceptible to practice effects [36,37] and is thus inappropriate for use in clinical trials as a primary endpoint. While many measures employ alternate forms, which can be beneficial, they are not immune to practice effects due procedural familiarity with the assessment process (e.g., knowing that a presented word list or visual display is likely subject to later recall). In addition to careful selection of measures, practice effects should be accounted for in the methodological design and statistical analyses. The significance of practice effects cannot be overstated, as they can significantly inflate Type I error rates by masking decline. Using an unreliable test leads to similar concerns.

In order to increase the potential for widespread adoption of an endpoint, the trial measures should also be readily available and easily accessible. Using measures that are difficult or expensive to obtain, and complicated and lengthy to administer will limit implementation. Identifying a small set of measures to be employed across FTD clinical trials will facilitate synthesis of results, meta-analysis and critical review, fostering development of a stronger evidence-base. With the increasing prevalence of multinational trials, using endpoints that have been translated and standardized across multiple languages is also beneficial where possible. The Addenbrooke Cognitive Examination, Revised (ACE-R) [38] and Montreal Cognitive Assessment (MoCA) [39] for example, have each been translated into several different languages facilitating international use.

## Global measures

As with RCTs for AD, clinical trials in FTD should give strong consideration for use of a combined measure that quantifies cognitive, behavioral and functional status in a single metric in order to increase sensitivity to change, particularly in the early phases of the disease. The Clinical Dementia Rating – Sum of Box Scores (CDR-SOB) is one such example that has been used in AD trials and an extension of the CDR adding two domains specific to FTD has also been developed (FTD-CDR), which includes ratings for Language as well as Behavior, Comportment and Personality [40]. The FTD-CDR has demonstrated an association with degree of hypometabolism on fluorodeoxyglucose positron emissions tomography (FDG-PET) studies [41] and demonstrated sensitivity to change in a mock clinical trial [40]. Similarly, the Clinician Global Impressions scales should also be considered, as they have already been implemented in several trials and have documented sensitivity to change [7]. The ACE-R, which incorporates the MMSE as well as further assessment of attention, memory, verbal fluency, language and visuospatial function has also shown sensitivity to change in bvFTD [42].

The CIBIC [4] is another example of a viable measure which incorporates a caregiver interview (CIBIC+). The CIBIC + utilizes Likert scales for disease severity and changes based on observation and written accounts summarizing semi-structured interviews evaluating behavior, cognition, and function and has demonstrated sensitivity to change in placebo groups [19]. Appropriate use of the FTD-CDR and CIBIC + relies on the expertise of the examiner and, as with any interview-based measure generating ratings on subjective input, being mindful of the quality and reliability of informant data is important. Training, clinical trial site quality, turnover of raters, and other operational details impact the quality of data collected and must be supervised in a RCT.

The sample size required to show a drug-placebo difference in a clinical trial depends on the observed rate of change, the standard deviation of the measure, and the effect size of the agent. The FTD-CDR changes by approximately 3.5 pointe per year. Anticipating a small effect size of disease-modifying agents (e.g., 25% showing), Knopman et al. (2008) estimated a sample size of 251 for an alpha of 0.05 and power of 80% (for a two arm trial). Composite scores based on multiple assessments of executive function or language function shows greater annual change and smaller sample sizes to demonstrate a drug benefit [40,43]. Recruiting the required number of patients will require multiple sites and diligent effort.

## Individual measures

For many reasons, a brief screening measure may be a tempting endpoint. However, selection of an appropriate measure becomes even more critical when using a brief measure with fewer items, as a smaller item pool negatively influences reliability and stability of estimates. The MMSE for example, has been used extensively as a screening tool and secondary outcome in clinical trials in AD, and has been one of the most frequent cognitive endpoints used in FTD trials to date. However, the MMSE lacks executive function measures and relies heavily on changes in memory to generate an abnormal score, which may not capture the cognitive changes in FTD. Not only does the MMSE have inadequate coverage of the target domains, it is also highly prone to ceiling effects and utilizes a relatively coarse metric, thus seriously limiting its appropriateness in a clinical trial setting. The MoCA may be a better alternative, showing increased sensitivity to cognitive impairment over the MMSE [44-46] while retaining a similar level of simplicity in both scoring and administration. The MoCA has demonstrated sensitivity to change over time in a dementia population [47]. The MoCA provides greater assessment of a broader range of cognitive abilities, including executive functioning and may capture critical elements of the FTD syndrome. The MoCA has been validated in multiple languages and has alternate forms available [48].

Targeted assessment of cognition, particularly language and executive functioning, may be warranted depending on the nature of the trial and study population. Assessment of language functioning is key for trials focusing on the language-predominant subtypes of FTD (i.e., semantic dementia, progressive non-fluent aphasia). Reliable assessment can be difficult due to the importance of qualitative changes (e.g., rate, prosody, latency) in language not readily captured by traditional language measures. In some instances it may be beneficial to generate audio recordings of participants to allow for multiple ratings of speech and language quality; however, quantitative metrics are needed. Two commonly employed clinical measures of expressive and receptive language that allow for flexibility in their administration and targeting of specific language components are the Western Aphasia Battery WAB; [49] and the Boston Diagnostic Aphasia Examination BDAE; [50]. The ACE has also demonstrated sensitivity to language impairments and change over time in PNFA and SD [51] and the Boston Naming Test BNT; [52] has also been widely used. Development and validation of novel assessment approaches and tools for measuring language may be required and advancements in voice recognition software and integration of technology may prove useful [53].

Given the known changes in frontal systems functioning, measuring executive functions should be an integral component of clinical trials in FTD. Trials in AD have previously employed trail making tests, fluency estimates, and response inhibition, though many of these tests are performance-based and vulnerable to practice effects, which will need to be prospectively addressed in the experimental design and data analysis. The Executive Interview (EXIT-25) is a brief cognitive screen that emphasizes executive function, and has been used in clinical trials in this population [7,54]. A similar executive screening measure, the Frontal Assessment Battery (FAB) [55], has been used with some suggestion of superiority to the EXIT-25 [56]. The NIH EXAMINER [33] is another battery developed explicitly as a brief, efficient method of assessing executive functions for use in clinical trials, however, multisite assessment and independent validation of this approach are needed.

Including assessment of memory is also important, though perhaps less so in comparison to AD trials where memory impairment is a primary symptom. If memory is to be quantified, selection of appropriate endpoints will require careful consideration, as traditional indices of memory functioning may be problematic as markers of cognitive change. Delayed free-recall scores are highly susceptible to floor effects, while recognition scores are limited by ceiling effects, particularly early in the phase of disease when changes are more likely to be very subtle. Alternatively, learning acquisition (i.e., learning over trials) as a marker of immediate recall, recall-recognition contrast measures, or recognition discriminability (i.e., hits vs. false-positives) may be better outcomes for assessing memory that are readily generated by many verbal and nonverbal list-learning tasks (e.g., California Verbal Learning Test, 2nd Ed.; [57]; Hopkins Verbal Learning Test; [58]).

## Composite measures

A potential risk of using multiple individual measures as the primary or secondary cognitive endpoints is the challenge of multiplicity, from which it may be difficult to derive meaningful change. Composite scores potentially address this issue by aggregating results from individual measures into a single cognitive index; however, use of composites must be theoretically justified. Creating a composite score via statistical data reduction methods (e.g., principal components analysis, factor analysis) may not be appropriate as it relies on *a posteriori* knowledge and capitalizes upon unique variances within the study sample that may limit generalization of the composites to other samples. A variant on generating a composite score is use of a standardized battery that generates both individual domain scores as well as a global index, which can be implemented across multiple sites using a common normative reference. In addition to the NIH EXAMINER, the cognitive subscale of the ADAS-Cog is an example of a composite battery that has been widely employed in AD drug trials. As with the MMSE, however, the ADAS-Cog targets the domains of memory and language and, in order to be appropriate for use in FTD trials, the expanded version, which includes additional assessment of executive functions, should be used [59]. Experience with this expanded version in FTLD is limited. The DRS and RBANS are two similar, brief cognitive batteries that have been used in clinical trials, however, neither provides adequate coverage of the executive domain and would need to be supplemented with additional measures. Another example of a composite measure designed and implemented in clinical trials for AD is the Neuropsychological Test Battery (NTB) [60]. The advantage of the NTB over other composites used in AD trials is the added focus on executive functioning and with known performance characteristics in clinical trials [61], it may be a viable endpoint for use in FTD trials.

## Behavioral measures

For trials targeting bvFTD, reliable assessment of behavioral functioning is an essential component. The NPI and Frontal Behavioral Inventory (FBI) [62,63] have both been shown to reliably differentiate between FTD subtypes at baseline [40] and have shown sensitivity to change over time [26]. In some circumstances, these measures may need to be supplemented with additional behavioral assessment tools due to their emphasis on more “positive” behavioral disturbances (e.g., agitation, irritability, disinhibition) over “negative” behaviors (e.g., apathy, indifference), which are among the core features of FTD. Including measures that capture more of these negative behaviors is recommended in order to ensure that the spectrum of behavioral disturbances is captured. The Frontal Systems Behavior Scale FrSBE; [64-66] is another option for quantification of behavioral disturbances that yields separate indexes for apathy, disinhibition and executive dysfunction. In addition to assessing apathy, the FrSBe also allows for intra-individual comparisons. A significant limitation with most, if not all, measures of behavioral disturbance is that they rely on the accuracy of caregiver reports. Integrating clinician ratings of behavior can be beneficial, however, these are restricted to observable behaviors that may not manifest in clinic and are heavily influenced by caregiver reports. Development of behavioral assessment methods that allow for greater objectivity and validation of caregiver reports may be particularly beneficial.

## Conclusions

Although not intended to be a comprehensive, nor exhaustive listing, Table 2, provides an overview of tools that could be considered for FTD trials, describing their roles, as well as potential strengths and limitations. Choosing appropriate endpoints for use in clinical trials is a complex and difficult decision that has direct implications on potential for success. For trials focusing on FTD, a principal challenge in choosing the optimal outcome measures will depend on how heterogeneous the targeted FTD sample is likely to be in a given trial. In studies focusing on one primary subtype (e.g., bvFTD), a primary outcome measure targeting that groups’ main symptom combined with a global or functional co-primary may be appropriate. Studies aimed at more heterogeneous samples on the other hand, may require outcomes surveying a broader range of functioning in order to generate meaningful results. Use of readily available measures that provide sufficient coverage of the targeted domain while retaining an adequate sensitivity to change is critical in order to maximize chances for beneficial outcomes. Development and application of appropriate trial outcomes is critically important to success in development of necessary treatments for FTD patients.

**Table 2:** Review of potential endpoints for consideration

**Domain**	**Test example**	**Strengths**	**Limitations**
Global	Clinician Interview Based Impression of Change (with caregiver interview)	Evaluates behavior, cognition and functioning; previously used in clinical trials; demonstrated sensitivity to change	Relies on subjective data from caregivers
	Clinical Dementia Rating	FTD-Specific version available; sensitive to change; association with biomarkers	Reliance on subjective data; lengthy to administer; coarse metric
	Clinical Global Impressions	Widely used in existing trials in FTD; sensitive to change; individual subscales available	Reliance on subjective data
Composite	Montreal Cognitive Assessment	Brief screen; sensitive to change; multicultural; alternate forms; freely available	Limited use in clinical trials; insufficient coverage of cognitive domains; potential for ceiling effects
	Repeatable Battery for the Assessment of Neuropsychological Status	Multi-domain assessment; alternate forms available	Inadequate coverage of executive functioning
	Dementia Rating Scale, 2nd Ed.	Multi-domain assessment sensitive to presence of dementia; previously used in clinical trials	Limited assessment of executive functioning; no alternate form
	EXAMINER	Developed with FTD in mind; intended for clinical trials; customizable; specific to executive functioning; measures social cognition and behavior	Actual trial performance is to be determined
	Neuropsychological Test Battery	Proven trial performance; sensitive to change	Relies heavily on memory functioning; no alternate forms
Executive	Trail Making Test	Previously used in clinical trials; extensive normative data; widely used	Limited sensitivity to change in previous trials; prone to floor effects
	Stroop Test	Multiple variants available; extensive normative data; previously used in clinical trials; relatively immune to ceiling effects	Sensitive to practice effects; interference conditions may be prone to floor effects
	EXIT-25	Previously used in FTD trials	Longer and more complicated administration than comparable alternatives
	Frontal Assessment Battery	Brief, simple administration; sensitive to change; multiple language versions	No alternate forms
	Clock Drawing	Sensitive to executive dysfunction; simple and brief administration; many variants available	Sensitivity and specificity vary as a function of version used.
Language	Boston Diagnostic Aphasia Examination	Sensitive to expressive and receptive language impairments;	Limited use in clinical trials; limited sensitivity to speech abnormalities; no alternate forms; prone to ceiling effects
	Western Aphasia Battery	Sensitive to expressive language impairments; previous use in clinical trials	limited sensitivity to speech abnormalities; no alternate forms; prone to ceiling effects
	Controlled Oral Word Association Test	Previously used in trials; sensitive to change	Only one well-validated alternate form; culturally limited
	Boston Naming Test	Widely used; extensive normative data; some use in trials	Non-normal distribution of scores; no alternate forms; culturally limited
Memory	California Verbal Learning Test, 2nd Ed.	Provides multiple estimates of memory (including learning) and insight into executive functioning	Only one alternate form; lengthy to administer; Recognition trials vulnerable to ceiling effects
	Rey Auditory Verbal Learning Test	Previously used in clinical trials; provides estimates of learning, recall and recognition	Recognition trials vulnerable to ceiling effects; recall trials vulnerable to floor effects
Visuospatial Functioning	Judgment of Line Orientation	Relatively free from practice effects; minimal demand on motor and language	Vulnerable to ceiling effects; can be lengthy administration
	Figure Copy tests	Insights into perception, organization and executive functioning; multiple forms	Confounded by motor impairment; scoring can be complex
Behavior	Neuropsychiatric Inventory	Widely used in clinical trials; sensitive to change	Not specific to behavioral changes associated with FTD; large standard variations; Improvements may be related to increasing apathy
	Frontal Behavior Inventory	Sensitive to change; employed in existing trials	Improvements may be related to increasing apathy
	Frontal Systems Behavior Examination	Allows for intra-individual comparison; quantification of apathy	Relies on reliable informant

## Competing interests

Dr. Cummings owns the copyright of the Neuropsychiatric Inventory. Drs. Miller, Banks and Leger declare that they have no competing interests.

## Authors’ contributions

All authors contributed to the preparation of this manuscript and have provided final approval of the version to be published.
